# Information-theoretic analysis of a dynamic release site using a two-channel model of depression

**DOI:** 10.1186/1471-2202-16-S1-P149

**Published:** 2015-12-18

**Authors:** Mehrdad Salmasi, Martin Stemmler, Stefan Glasauer, Alex Loebel

**Affiliations:** 1 Graduate School of Systemic Neurosciences, Ludwig-Maximilian University, Munich, Germany; 2 German Center for Vertigo and Balance Disorders, Ludwig-Maximilian University, Munich, Germany; 3 Bernstein Center for Computational Neuroscience, Munich, Germany; 4 Department of Biology II, Ludwig-Maximilian University, Munich, Germany; 5 Department of Neurology, Ludwig-Maximilian University, Munich, Germany

## 

Synapses are dynamic communication channels between neurons as their rates of information transfer depend on past history. While information theory has been used to study the information efficacy of synapses [1-3], the effect of synaptic dynamics, including short-term depression and facilitation, on the information rate is not yet fully understood.

**Figure 1:**
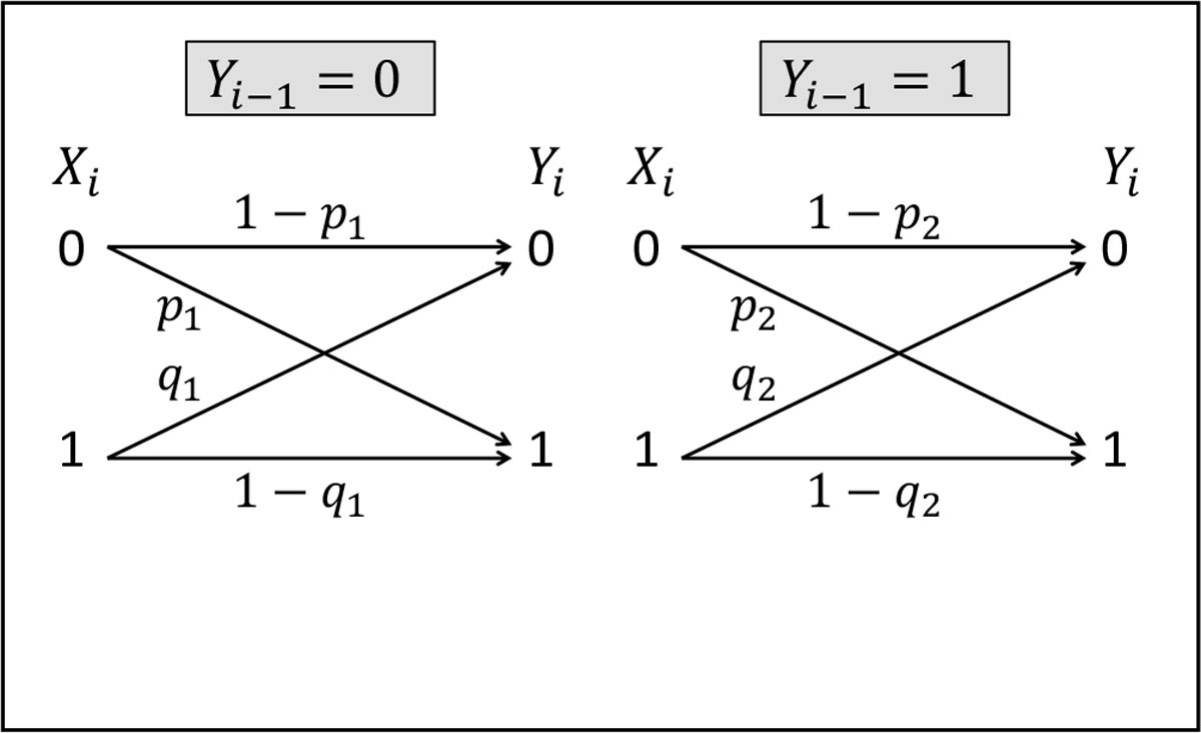


To reduce the complexity of the problem, we confine ourselves here to a single release site of the synapse. This allows us to analytically calculate the information transfer at the release site for a simple model of synaptic depression which is based on binary channels. The input of the model is a spike train, modeled by an independent identically distributed process $\text{X}={\left\{{\text{X}}_{\text{i}}\right\}}_{\text{i}=1}^{\infty }$, where each *X_i _*has a Bernoulli distribution with *P*(*X_i _*= 0) = α. The model's output is a process $\text{Y}={\left\{{\text{Y}}_{\text{i}}\right\}}_{\text{i}=1}^{\infty }$, such that if there is a release at time, then *Y_i _*= 1 and otherwise *Y_i _*= 0. We model the short term depression by two binary asymmetric channels that represent the possible states of the release site: the 'recovered' state, when no release occurred in the previous time step (Figure 1a), and the 'used' state, following vesicle release (Figure 1b). In particular, we assume that the release probability is reduced following a release, that is *p*_2 _≤ *p*_1 _and 1 - *q*_2 _≤ 1 - *q*_1_.

Each individual channel in Figure 1 will have a mutual information rate, either *r_1_* or *r_2_*. As *X_i_* is Bernoulli-distributed, ${\text{r}}_{\text{i}}=\text{h}\left(\alpha {\text{p}}_{\text{i}}+\bar{\alpha }\bar{{\text{q}}_{\text{i}}}\right)-\alpha \text{h}\left({\text{p}}_{\text{i}}\right)-\bar{\alpha }\text{h}\left(\bar{{\text{q}}_{\text{i}}}\right)$ for *i *= 1,2, where *h*(·) is the entropy of a Bernoulli random variable and $\bar{\text{x}}=1-\text{x}$. We prove that the mutual information rate of the release site with depression is a linear summation of the information rates of these two channels. The mutual information rate *I(X;Y) *between the input process *X *nd the output process *Y*, is *I(X;Y) = θr_1 _+ (1 - θ)r_2 _*where $\theta =\frac{\alpha \bar{{\text{p}}_{2}}+\bar{\alpha }{\text{q}}_{2}}{\alpha \left({\text{p}}_{1}+\bar{{\text{p}}_{2}}\right)+\alpha \left(\bar{{\text{q}}_{1}}+{\text{q}}_{2}\right)}$

The closed form expression of the mutual information rate allows us to study the effect of depression analytically. Through simulations we show that for a range of parameters, depression improves the rate of information transfer at the release site. We also show that when the level of depression is increased (i.e., with smaller *p*_2 _and larger *q*_2_), the release site's information capacity is reached at lower input spike rates. Therefore, the optimal spike rate of the presynaptic neuron has a reverse relationship with the depression level of its release site. This means that synaptic depression can save energy while maintaining information rate. The two-channel model of release site is a building block for the construction of more precise models of synaptic transmission. These advanced models will enable us to evaluate and study the synaptic information rates analytically.
